# Dynamic Modeling of Pesticide Residue Determination to Ensure Safe Food: A Review

**DOI:** 10.3390/foods15050798

**Published:** 2026-02-24

**Authors:** Shelim Mohammad Jahangir, Kaniz Fahima Rova, Md. Intesar Farhan Labib, M. A. A. Shoukat Choudhury, Mohammad Dalower Hossain Prodhan

**Affiliations:** 1 Department of Chemical Engineering, Bangladesh University of Engineering and Technology, Dhaka 1000, Bangladesh; kanizrova@gmail.com (K.F.R.); intesarfarhanlabib@gmail.com (M.I.F.L.); shoukat@che.buet.ac.bd (M.A.A.S.C.); 2 Pesticide Research & Environmental Toxicology Section, Entomology Division, Bangladesh Agricultural Research Institute (BARI), Joydebpur, Gazipur 1701, Bangladesh; mdhprodhan@gmail.com

**Keywords:** pesticide, modeling, analytical model, semi-empirical model, empirical model, discussion articles

## Abstract

The modeling of pesticide phenomena aids researchers and policymakers in reaching complex agricultural decisions. A contemporary global concern among consumers revolves around the presence of pesticide residues in food, which possesses significant threats to human health and non-target organisms. Throughout all parts of the world, pesticide concentration in food crops is determined by laboratory analysis. In a limited number of areas, predictions of pesticide concentration are performed by analytical or numerical modeling. In this study, a thorough review of pesticide models for predicting their concentrations is critically performed. Among the 53 papers examined in this study, 31 of them are based on a theoretical concept, four amalgamate empirical data with a theoretical background, ten of them are based on only experimental results, and lastly, eight are discussion articles describing observations from various field studies. Overall, more than 69% of the papers focus on direct pesticide–plant interactions which affect our food chain, while indirect effects on environmental components, especially surface water, also receive attention. To the best of our knowledge, among the limited reviews, this is the first to attempt to accumulate all such modeling information in one place, critically analyze all papers to identify their limitations and scopes, and finally provide future research directions in this area.

## 1. Introduction

The growth of the world’s population has been supported by a corresponding increase in food production, and pesticides play a crucial role by maintaining stable crop yields. As a result, an estimated one-third of global agricultural products are produced with the assistance of pesticides [1]. These compounds are cost-effective, labor-saving, and highly efficient in controlling pests and diseases. Therefore, they are widely used during crop production, storage, transportation, and marketing to protect agricultural commodities and reduce economic losses. Because of their protective function, pesticides are also referred to as Plant Protection Products [2].

Pesticides are applied not only in agriculture but also in forestry, public parks, gardening, and household settings. Moreover, each formulation contains at least one active substance—defined as a chemical, plant extract, pheromone, or microorganism (including viruses) that acts against pests or affects plants or plant products [3]. Their functions include protecting crops from pests and diseases both before or after harvest, as well as influencing plant growth processes (excluding nutrients), in addition to preserving plant products and controlling unwanted vegetation.

Despite their benefits, pesticide residues in agricultural products pose potential toxicological risks. Within the framework of Life Cycle Analysis [4], it is essential to evaluate the fate of pesticides in the environment, their persistence in crops, and the resulting human exposure. In this regard, exposure may occur through direct contact with contaminated produce or through dietary intake. Consequently, public concern regarding pesticide residues in food has increased due to their possible adverse health effects. In general, human exposure typically occurs at low levels over long periods via food, air, and water. However, while most toxicological evidence comes from animal studies using doses higher than those permitted in food, occasional short-term exposure to higher residue levels may occur due to consumption patterns or variability among food products.

Beyond human health concerns, pesticides can negatively affect the environment by contaminating soil, water, and air and as well as by harming non-target organisms. Improper application has been associated with biodiversity loss, pollution from spray drift and runoff, injury to beneficial species, and damage to crops when used excessively or incorrectly. Non-target organisms such as pollinators, aquatic species, birds, and amphibians are particularly vulnerable [5]. For example, neonicotinoid insecticides have been linked to pollinator decline, posing risks to ecosystem services and food security.

To minimize these risks, Integrated Pest Management (IPM) has therefore been promoted as a sustainable approach to pesticide use. In this approach, IPM emphasizes biologically effective, user-friendly, environmentally safe, economically viable, and innovative pesticides [6]. Proper application practices, including correct timing, dose adjustment based on pest pressure, and improved agronomic techniques, further reduce pesticide dependency. Importantly, pesticide use indicators such as active ingredient quantity or cost should be interpreted cautiously, because they do not directly reflect environmental impact; safer and more innovative products are often more expensive.

Recent advances in chemistry, biology, and molecular sciences have significantly improved the understanding of pesticide behavior, residue dynamics, and environmental risks. Consequently, modern pesticides with safer profiles and improved application technologies have emerged. Mathematical and empirical models are increasingly used to predict pesticide translocation, residue persistence, and their environmental fate within soil–plant–atmosphere systems. Although these models vary in complexity, they provide essential tools for assessing pesticide behavior, supporting risk evaluation and guiding sustainable crop protection strategies.

The main objectives of this review study are:

(i) To critically assess current pesticide models’ merits and drawbacks, find information/data gaps, and identify areas that need additional research.
(ii) To assist in the integration of available knowledge from the literature that will help to establish a safe, sustainable usage of pesticide in agriculture.
(iii) To elucidate the underlying ideas of pesticide fate, transport, and effects in soil, water, air, and biota.
(iv) To point out the possible directions for further study on pesticide modeling, such as boosting the resilience of the models and making them more easily accessible.

## 2. Materials and Methods

The authors designed and rigorously followed a structured review methodology based on an iterative process that involved identifying appropriate search keywords, selecting relevant studies, and subsequently conducting the analysis. The methodology was developed in alignment with the central research question and the formulated search string to ensure the extraction of pertinent studies. All authors collaboratively defined and developed the required stages of the methodology. Publication types were limited to peer-reviewed journal articles and crop residue and pesticide fate modeling studies; reviews and non-English papers were excluded, duplicates were removed, and finally 53 papers were included.

## 3. A Brief History of Pesticide Modeling

One of the earliest pioneers of agricultural system modeling was C. T. de Wit, who demonstrated that agricultural systems could be quantitatively described by integrating fundamental physical and biological principles [7]. His work emphasized energy balance, mass transfer, and plant physiological processes, laying the foundation for mechanistic crop modeling. Conducted primarily at Wageningen University, de Wit’s research transformed agricultural science from a largely empirical discipline into a predictive, equation-based field capable of simulating complex biological systems.

Another influential contributor was W. G. Duncan, whose pioneering simulation studies on canopy photosynthesis examined the interactions among light interception, carbon assimilation, and plant architecture [8]. Duncan’s work significantly advanced understanding of crop growth dynamics and provided the basis for later crop growth and yield models. Together, these early efforts demonstrated the potential of modeling to improve agricultural management and decision-making.

As agricultural modeling matured, its scope expanded beyond crop growth to encompass environmental impacts, particularly the fate and transport of pollutants such as pesticides. A major milestone was the work of [9], who developed an integrated model to assess pollutant fate across multiple environmental media. This approach facilitated the incorporation of agricultural emissions into life cycle assessment (LCA) frameworks by tracking pesticide distribution among soil, water, and air compartments.

Building on this foundation, Fantke, Juraske, and colleagues [10] reviewed a range of crop-specific pesticide uptake models describing chemical absorption, translocation, degradation, and accumulation within plants. Most of these models rely on first-order kinetics with constant coefficients, assuming proportional relationships between pesticide concentrations and transport or transformation rates. While computationally efficient, such assumptions may oversimplify the complex and variable conditions of real agricultural systems.

Pesticide fate in crops is strongly influenced by physicochemical properties such as solubility, vapor pressure, and partition coefficients, as well as by crop morphology and physiology. These processes vary substantially among plant species and cultivars [11] and are further affected by environmental factors including temperature, soil moisture, radiation, and microbial activity. Consequently, realistic uptake models must simultaneously account for chemical properties, environmental conditions, and crop-specific characteristics.

Because agricultural systems are inherently dynamic, steady-state assumptions are often invalid. Weather variability, plant growth, and management practices prevent equilibrium from being achieved, necessitating dynamic modeling approaches. These approaches involve solving time-dependent differential equations describing pesticide concentrations across interconnected compartments [4], providing improved representations of short-term exposure and peak concentration events.

Several dynamic, crop-specific models have been developed to address these challenges. Trapp and Legind and Rein [12] proposed mechanistic models for cereals incorporating growth, transpiration, and chemical transport, while Juraske [13] developed dynamic models for vegetables. Despite their enhanced realism, such models require numerous chemical- and crop-specific parameters, many of which are uncertain or unavailable, limiting their practical applicability [14].

Subsequent developments include refined emission models such as PestLCI 2.0 [15], regulatory tools like SWASH for EU surface water assessments [16], and recent empirical and semi-empirical approaches [17,18]. Collectively, these advances reflect a clear progression toward dynamic, integrated, and application-oriented pesticide fate modeling frameworks that support sustainable and safe food production.

## 4. Classification of the Models and Studies

A comprehensive survey of the scientific literature identified 53 research papers directly relevant to the modeling of pesticide residues in agricultural systems. These studies were systematically screened and classified according to their methodological approaches, underlying assumptions, and the types of data used for model development. This classification provides a structured overview of the evolution, diversity, and applicability of pesticide residue modeling approaches, highlighting their respective strengths and limitations in addressing food safety concerns.

A subset of the reviewed studies relied exclusively on established theoretical principles without incorporating experimental or field-derived data. These studies are categorized as analytical models. Analytical models are based on physicochemical laws such as mass-balance equations, degradation kinetics, diffusion, and transport processes. Their primary advantages include conceptual clarity, mathematical transparency, and broad applicability across different scenarios. However, the lack of empirical validation may limit their ability to accurately represent real agricultural systems, where environmental variability and biological interactions strongly influence pesticide behavior.

Another group of studies employed a hybrid methodology that integrates theoretical formulations with experimental or field observations, referred to here as semi-empirical models. These models seek to bridge the gap between idealized theory and practical reality by calibrating model parameters using measured data. Semi-empirical models are particularly valuable for pesticide residue studies because they can incorporate crop physiology, application practices, climatic conditions, and soil properties. While these models often offer improved predictive performance, their reliability depends heavily on the quality, representativeness, and scale of the empirical data used for calibration and validation.

A third category consists of empirical models developed entirely from experimental measurements or observational datasets. These models rely on statistical relationships rather than explicit mechanistic descriptions. Empirical models are especially effective for predicting pesticide residue levels under specific crops, regions, or management practices, making them useful for regulatory assessments and decision support. However, their limited transferability across different environmental conditions or cropping systems restricts their broader applicability.

In addition to model-based studies, several papers focused primarily on field observations, experimental findings, or qualitative assessments without proposing explicit modeling frameworks. These are classified as discussion articles. Although they do not provide predictive tools, such studies offer essential insights into pesticide degradation, residue persistence, exposure pathways, and environmental interactions, thereby informing future model development.

To ensure a comprehensive assessment, this review also considered studies addressing indirect pesticide effects through environmental compartments such as soil, water, air, and non-target organisms. These components can act as secondary sources of residues that re-enter crops and the food chain. By integrating both direct and indirect pathways, this review offers a holistic perspective on pesticide residue modeling, supporting the advancement of robust predictive tools for food safety and environmental sustainability.

Based on these observations, the currently available models and studies can be classified as follows in Table 1:

Here, the direct effect models deal with the impact of pesticide application in different compartments of the plant which enter our food chains. The indirect effect models deal with the environmental impacts resulting from pesticide application. The following section illustrates each category of models.

### 4.1. Analytical Models

Analytical models are primarily developed from the theoretical foundations of fundamental science and engineering principles. They rely on first-principle formulations, including mass-balance equations, transport phenomena, and mass transfer theories, to describe the behavior and fate of pesticides within agricultural and environmental systems. By applying established physical and chemical laws, these models provide a mechanistic understanding of pesticide dynamics rather than depending solely on empirical observations. Consequently, analytical models are often regarded as transparent, robust, and transferable across different systems, provided that their underlying assumptions remain valid.

In pesticide residue research, analytical models are commonly used to simulate processes such as pesticide deposition, diffusion, volatilization, degradation, and uptake by plants or surrounding environmental media. Compared with empirical approaches, these models typically require fewer experimental data inputs; however, they demand accurate parameterization and a solid theoretical framework. Table 2 presents a comparative summary of the available analytical models, outlining the crops investigated, pesticides applied, areas of application, and key limitations associated with each modeling approach. This overview assists researchers in selecting appropriate models for specific crops or environmental conditions while recognizing their constraints.

Based on their focus, analytical models can be broadly classified into direct effect and indirect effect models. Direct effect models explicitly describe pesticide–plant interactions, including foliar deposition, absorption through plant surfaces, internal translocation, and residue accumulation in edible tissues. These models are particularly valuable for predicting pesticide residues in food crops and evaluating potential risks to food safety. In contrast, indirect effect models examine pesticide behavior in surrounding environmental compartments such as soil, water, and air. They address processes including leaching, runoff, volatilization, and atmospheric dispersion, thereby supporting assessments of environmental contamination and ecological risk. Despite their strengths, analytical models are often limited by simplifying assumptions, spatial homogeneity, and insufficient representation of biological variability, which may reduce their predictive accuracy under complex field conditions.

#### 4.1.1. Direct Effect Analytical Models

Direct effect analytical models are designed to evaluate the immediate impacts of pesticide applications within different plant compartments. These models focus on the transport, uptake, distribution, and persistence of pesticide residues inside plant tissues following application. For systematic analysis, direct effect models are commonly classified into five categories according to the plant compartment considered: root analyzer models, stem analyzer models, leaf analyzer models, fruit and seed analyzer models, and aerial parts analyzer models. Each category employs specific assumptions and mathematical formulations to capture the dominant mechanisms governing pesticide behavior within that compartment.

Root analyzer models describe pesticide uptake and accumulation in plant roots, which often represent the primary entry pathway for soil-applied or soil-resident chemicals. Trapp [19] developed a dynamic uptake model based on diffusion and partitioning principles to simulate the absorption of neutral lipophilic compounds from the soil into roots. Dettenmaier [20] further advanced this approach by establishing a relationship between the transpiration stream concentration factor (TSCF) and the octanol–water partition coefficient (log K_o_w), demonstrating that neutral polar compounds exhibit enhanced root uptake. More recently, Jorda et al. [21] proposed a mechanistic mass-balance model explicitly accounting for soil–root interactions and pesticide physicochemical properties.

Stem analyzer models focus on pesticide transport and redistribution within stems, which act as conduits between roots and aerial tissues. Xiao et al. [22] applied a radial diffusion-based model to describe pesticide migration within a spherical potato specimen, enabling prediction of internal concentration gradients. In contrast, Juraske et al. [23] incorporated temporal factors such as application-to-harvest intervals and soil degradation processes, linking stem residues to environmental and management conditions.

Leaf analyzer models estimate pesticide retention on foliage and emissions to air, soil, and water. PestLCI [24] and its updated version PestLCI 2.0 [15] provide widely used life cycle inventory frameworks. Hwang et al. [25] further developed a lettuce-based model integrating deposition, dissipation, and translocation to predict residues in edible leaves.

Fruit and seed analyzer models examine pesticide translocation and persistence in edible reproductive tissues. Juraske et al. [57] and Li [18] modeled residue dynamics in fruits under varying agronomic and environmental conditions, supporting MRL compliance. Aerial parts analyzer models integrate aboveground compartments; notable contributions include the framework by Fantke et al. [10], later enhanced by Rein et al. [12], and the foundational mass-balance formulation of Trapp and Matthies [26].

#### 4.1.2. Indirect Effect Analytical Models

Indirect effect analytical models address the impacts of pesticides on plants arising from residues in surrounding environmental compartments rather than from direct application to crop surfaces. These models focus on secondary exposure pathways through which pesticides may re-enter plant systems over time via air, water, or soil. Such indirect exposure can lead to long-term agronomic and ecological consequences, as residues may cycle back into cultivation zones through irrigation, soil moisture, runoff, leaching, or atmospheric deposition. Consequently, indirect effect models are essential for understanding the persistence, transport, and delayed effects of pesticides within agroecosystems.

For analytical convenience, indirect effect models are commonly classified into water analyzer models and soil analyzer models, depending on the environmental medium considered. Water analyzer models evaluate pesticide transport to and accumulation in surface water bodies, while soil analyzer models focus on pesticide fate within the soil–plant continuum, including root uptake and potential groundwater contamination.

Among water analyzer models, SWASH (surface water scenarios help), developed by van den Berg, Beltman, and Adriaanse [40], is a widely applied analytical tool within the European Union regulatory framework. SWASH estimates pesticide exposure concentrations in surface waters under standardized EU FOCUS scenarios by integrating application data with environmental processes such as runoff, drainage, spray drift, and degradation. The model plays a key role in regulatory risk assessment by providing transparent and harmonized exposure estimates.

To address protected cultivation systems, Wipfler et al. [64] developed a greenhouse emission model to quantify pesticide losses to surface water from greenhouse crops grown on substrates under open and closed systems. This model accounts for irrigation practices, recirculation efficiency, substrate characteristics, and drainage losses, enabling improved assessment of the environmental risks associated with greenhouse agriculture.

Soil analyzer models form another major subgroup, examining pesticide sorption, degradation, leaching, and root uptake. A prominent example is the PEARL model [41], a one-dimensional numerical model widely used in Europe to assess groundwater contamination and indirect crop exposure. Earlier studies, such as Behrendt et al. [42], further demonstrated how soil-based models can elucidate pesticide transport into plant transpiration streams.

### 4.2. Semi-Empirical Models

Semi-empirical models occupy an important intermediate position in pesticide residue modeling by combining the strengths of theoretical (analytical) and data-driven empirical approaches. These models are built on sound theoretical foundations—such as mass-balance principles, kinetic equations, and transport processes—while incorporating experimental or field-based data to calibrate and validate model parameters. As a result, semi-empirical models are generally more realistic than purely analytical models and more transferable than strictly empirical ones. Representative examples and their applications are summarized in Table 3.

Based on their focus, semi-empirical models can be broadly classified into direct effect and indirect effect models. Direct effect semi-empirical models explicitly address pesticide behavior within plant compartments, including leaves, stems, roots, or aerial parts. They describe processes such as uptake, translocation, degradation, and metabolism by combining theoretical kinetics with experimentally derived rate constants. An illustrative example is the aerial-part analyzer developed by Li and Fantke [31], which estimates pesticide degradation rate constants in potato plants. By integrating experimental data with theoretical degradation models, this approach improves predictions of pesticide dissipation under realistic growth and environmental conditions, supporting pre-harvest interval determination and compliance with maximum residue limits.

Indirect effect semi-empirical models focus on pesticide behavior in environmental compartments rather than within crops. These models assess off-target transport, transformation, and ecological impacts in systems such as surface water, soil, and groundwater. A notable example is the MASTEP model [46], which combines toxicokinetic theory with experimental ecotoxicological data to simulate the effects and recovery of aquatic organisms following pesticide exposure. Another example is the cascade-based runoff model developed by Wallach [47], which applies mass-balance principles and empirical parameterization to simulate pesticide concentrations in surface runoff.

Finally, GLEAMS [48] represents a widely used semi-empirical model for evaluating pesticide leaching to groundwater. Although primarily comparative in nature, it is highly valuable for assessing management practices and environmental risk. Overall, semi-empirical models effectively bridge theory and observation, enhancing pesticide risk assessment and supporting sustainable agriculture.

### 4.3. Empirical Models

Empirical models are developed by fitting mathematical relationships directly to experimental or observed data, without explicitly describing the underlying physical, chemical, or biological mechanisms. These models are particularly valuable when system complexity, limited data availability, or incomplete theoretical understanding restrict the application of analytical approaches. In pesticide residue research, empirical models rely on field trials, laboratory experiments, and monitoring datasets to characterize the behavior, distribution, and persistence of pesticide residues under specific agricultural conditions. Their predictive accuracy depends strongly on the quality, quantity, and representativeness of the data used for calibration and validation.

Empirical models can be broadly categorized into direct effect and indirect effect models. Direct effect empirical models focus on pesticide residues within plant compartments such as roots, stems, leaves, fruits, and seeds. These models are widely applied to estimate residue dissipation rates, translocation patterns, and pre-harvest intervals, thereby supporting food safety evaluations and regulatory decision-making. Because they are closely aligned with observed data, direct effect empirical models often provide reliable predictions within the conditions for which they were developed.

Indirect effect empirical models address pesticide residues in surrounding environmental compartments, including soil, water, and air, and their secondary influence on crops. These models capture processes such as leaching, runoff, volatilization, and re-deposition, which can indirectly contribute to crop contamination over time. While empirical models are generally limited in their transferability beyond the conditions under which they were developed, they remain practical and effective tools for predicting pesticide residue behavior in real agricultural settings.

#### 4.3.1. Direct Effect Empirical Models

Direct empirical models describe pesticide behavior within plant systems using relationships derived from experimental observations. These models are classified into fruit, leaf, stem, vegetative parts, and aerial parts analyzer models, depending on the plant compartment studied. Each category examines the accumulation, transport, transformation, and dissipation of residues, providing a detailed understanding of exposure risks.

Fruit analyzer models focus on pesticide uptake, translocation, and degradation in fruits, which are critical for dietary exposure. Fantke et al. [49] used multi-compartment modeling to separate uptake, translocation, degradation, and volatilization processes. Hlihor et al. [30] linked residue decline to human exposure, while An et al. [50] integrated soil–plant interactions to simulate residue levels in fruits, roots, stems, and leaves.

Leaf analyzer models address pesticide fate on and within leaves, capturing deposition, wash-off, and degradation. Das et al. [51] highlighted limitations in predicting semi-volatile pesticide emissions, emphasizing the need to consider volatilization and atmospheric exchange.

Stem and vegetative parts analyzer models evaluate transport and retention in stems, shoots, and other vegetative tissues. Gopalakrishnan et al. [52] showed that stem composition affects sorption and diffusion, while Hwang and Moon [65] linked soil residues to uptake in vegetative tissues.

Aerial parts analyzer models treat aboveground components collectively, supporting harvest-time residue assessments. Charles [4] and Xiao et al. [66] demonstrated the influence of plant morphology on residue accumulation. Collectively, these models translate experimental data into practical tools for residue prediction and food safety assessment.

#### 4.3.2. Indirect Effect Empirical Models

Indirect empirical models focus on pesticide behavior in environmental compartments—soil, water, and air—rather than directly within plant tissues. These models are essential for understanding off-target exposure, environmental fate, and ecological risks of pesticide applications.

Early models, such as that by Asman et al. [54], quantified pesticide runoff and drift to adjacent water bodies, incorporating spatial factors like the distance from treated fields. Such models highlighted the influence of landscape configuration on pesticide contamination dynamics. Indirect empirical models have also been integrated into life cycle assessment (LCA) frameworks. Peña et al. [17] developed a model linking pesticide emission data with LCA impact assessments, enabling temporal separation of emission events and a more accurate evaluation of environmental impacts.

Other studies investigated the operational and environmental factors affecting deposition on soil and plant surfaces. Bahrouni et al. [55] showed how nozzle type, pressure, and wind speed influence pesticide distribution. Kulhánek et al. [56] examined bioconcentration in edible plant tissues.

Overall, indirect empirical models provide data-driven insights into the environmental transport, transformation, and secondary exposure pathways of pesticides. By complementing direct empirical approaches, these models improve risk assessments, guide regulatory policies, and support sustainable pesticide management.

A summary of the empirical models is represented in Table 4 along with the type of plant implemented for the experiment, the pesticides used, their applications, and their limitations.

### 4.4. Discussion Articles

Discussion articles synthesize previous research, identify knowledge gaps, and guide future studies in pesticide residue modeling, as summarized in Table 5. They provide critical insights into pesticide accumulation and variation across plant compartments under diverse environmental and agronomic conditions, helping to compare and evaluate different modeling approaches.

Some discussion articles focus on theoretical foundations, evaluating models for neutral compounds versus weak or strong electrolytes [58] and clarifying assumptions, limitations, and applicability. Others examine pesticide residues in specific plant parts, including non-edible tissues, to assess environmental and ecological risks [59]. Meta-analyses, such as those by Keikotlhaile et al. [60], investigate the effects of food processing on residue levels, while studies on photodegradation [61] highlight natural dissipation mechanisms.

Several articles address human health impacts by assessing dietary exposure to pesticide residues, considering regional differences in consumption patterns and regulatory frameworks [57]. Others explore indirect ecological effects, using models like PRIMET to estimate risks to non-target organisms [62] or integrating life cycle assessment approaches to evaluate human and ecosystem impacts [9].

Overall, discussion articles bridge theoretical, empirical, and practical perspectives, guiding improvements in modeling, risk assessment, and sustainable agricultural practices. They play a pivotal role in advancing knowledge and shaping future pesticide research.

## 5. Future Research Directions

In this study, a thorough review of the evolution of pesticide models from their initial conceptualizations to the current integrated models is provided. The applications and drawbacks of current pesticide models, and identification of the gaps and limitations of the current models that require further research, are also critically assessed.

To date, no model has successfully integrated all factors associated with plants and the environment. Many existing models have overlooked indirect factors, assuming that plants only absorb applied pesticides and have not considered potential contamination from pesticides already present in the soil or water. Simultaneous consideration of the effects of both direct and indirect factors could enhance the realism of future models. Moreover, current models predominantly focus on specific crops and pesticides, so the exploration of additional combinations is strongly needed. Specifically, for Bangladesh, it is essential to model important vegetables like brinjal and cauliflower with suitable pesticides. Additionally, the majority of existing models have primarily dealt with neutral organic pesticides, so models with ionic pesticides should be highlighted in the future.

More precisely, among the models, the most valid, flexible and popular one till today is ‘New concepts for dynamic plant uptake models’ [12]. However, this model also has some serious limitations. In case of diffusive mass transfer consideration, it assumes that the concentration in a compartment’s water content is equal to that of the dry portion. Instead, there should be an equilibrium distribution of pesticides between the dry part and the moisture part as they are two different phases. This model also does not consider the entry of pesticides into the air after application to the soil. Lastly, this model assumes the roots’ radius will be constant throughout the growth stage. However, the roots’ radius should increase with growth.

## 6. Conclusions

This literature review provides a comprehensive assessment of research addressing the quantification and dynamics of pesticide residues in plants and their interactions with the surrounding environment. Based on a critical analysis of 53 selected studies, the review identifies prevailing research trends, methodological approaches, and existing knowledge gaps related to pesticide behavior, dissipation, and environmental interactions. The findings highlight complex linkages among plants, soil, air, and water, underscoring their importance for food safety, environmental sustainability, and agricultural management.

Among the reviewed studies, 31 were purely theoretical, focusing on mechanistic descriptions of pesticide transport, translocation, and degradation using principles such as mass balance, diffusion, and kinetic modeling. Four studies adopted hybrid approaches that integrated empirical data with theoretical frameworks, enhancing model realism and predictive capability. Eleven studies were experimental, generating laboratory-based data on residue accumulation and dissipation in different plant compartments, while eight relied on field observations or secondary data analyses, reflecting real-world agricultural conditions.

Most of the studies (37 out of 53) emphasized direct pesticide transport and accumulation within plant compartments, particularly the aerial parts relevant to human consumption. Indirect effects, especially surface water contamination, were less frequently studied but remain critical due to ecological and regulatory concerns. Overall, the literature highlights the need for integrated modeling, experimental, and field-based approaches to better understand pesticide fate, support risk assessment, and promote sustainable agricultural practices.

## Figures and Tables

**Table 1:** Classification tree of the current pesticide residue modeling works. (First, the research papers are grouped into four categories, namely analytical models, semi-empirical models, empirical models and discussion articles. Each of these categories are further classified into direct effect and indirect effect models).

Study Type	Effect Type	References
Analytical	Direct Effect	[10,12,13,15,19,20,21,22,23,24,25,26,27,28,29,30,31,32,33,34,35,36,37,38,39]
	Indirect Effect	[40,41,42,43,44,45]
Semi-Empirical	Direct Effect	[18]
	Indirect Effect	[46,47,48]
Empirical	Direct Effect	[4,49,50,51,52,53]
	Indirect Effect	[17,54,55,56]
Discussion Articles	Direct Effect	[57,58,59,60]
	Indirect Effect	[9,61,62,63]

**Table 2:** Existing analytical models that estimate residues of pesticides in different parts of plants are shown below.

	Pesticide Model Name/Journal Paper Name	Plant for Experiment	Pesticides Used	Application of the Models	Limitations	Reference
**Direct Analytical Models**						
Root Analyzer	Dynamic Root Uptake Model for Neutral Lipophilic Organics	Carrot	Benzo[a] pyrene, polychlorinated biphenyls (PCBs), and chlorobenzenes	Estimating pesticide residue in different compartments of a plant.	This model overestimates the result for thick roots whereas it underestimates the result for leaves.	[19]
	Measuring and modeling of root uptake of organic chemicals	Soybean, tomato	1,4-dioxane, MTBE, and sulfolane	Predicting the final plant tissue concentrations for use in risk assessment analysis.	Difficulties with measuring uptake variables for a clearer solution.	[20]
	Mechanistic modeling of pesticide uptake with a 3D plant architecture model	Maize crop	Any pesticide	This model aims to simulate the absorption of pesticides from the soil and scrutinize the factors influencing this process such as the impact of root and substance properties.	Its limitation to neutral compounds when simulating large root architectural systems, and the laborious parameterization of root hydraulics remain a challenge.	[21]
Stem analyzer	Improving Pesticide Uptake Modeling into Potatoes: Considering Tuber Growth Dynamics	Potato	Chlorpyrifos	Predicting chlorpyrifos in potato at harvesting time.	There is limited information regarding pesticide residues in sprouting potatoes.	[22]
	Pesticide Uptake in Potatoes: Model and Field Experiments	Potato	Chlorpyrifos	Predicting the magnitude and temporal profile of the experimentally derived pesticide concentrations.	Tubers are considered to be homogeneous mixed spheres. Actually, this is not true.	[23]
	Sorption of Lipophilic Organic Compounds to Wood and Implications for Their Environmental Fate	Oak and basket willow	Phenol, benzene, chlorobenzene, o-xylene, 1,2-DCB, lindane, naphthalene, 1-3,5-TCB dieldrin, DDT	Identifying the sorption of lipophilic organic compounds to wood and the implications for their environmental fate	Degradation rates and pathways of xenobiotics in wood are unknown.	[27]
Leaf Analyzer	PestLCI—A model for estimating field emissions of pesticides in agricultural LCA	Cereal, pear, orange	Bentazone, MCPA, pendimethalin	Estimation of field emission of pesticides in different compartments of the environment.	This model is prepared to incorporate variations of all of the system parameters but there is a need to establish the relevant data for countries other than Denmark, where the model was developed.	[24]
	PestLCI 2.0 a second-generation model for estimating emissions of pesticides from arable land in LCA	Maize, potato	MCPA, bentazone	Estimating emissions of pesticides at different arable land areas.	Data availability is limited.	[15]
	Comparison of Theoretical and Experimental values for plant uptake of pesticide from soil	Lettuce	Chlorpyrifos	Estimating pesticide residue in different compartments of a plant.	The desorption and leaching of pesticides in soil, the landscape of the field, environmental weather, and contact area between plant roots and soil are considered.	[25]
	A modeling approach to study the pesticide dynamics to reduce pesticide residues in Japanese green tea	Green tea	Azoxystrobin, clothianidin	Reducing the residue levels in green tea and proposing an alternative GAP.	Sufficient field data were not available.	[28]
Fruit and Seed Analyzer	Uptake and persistence of pesticides in plants: Measures and model estimates of imidacloprid after foliar and soil application	Tomato	Imidacloprid	Estimating the time-dependent contaminant concentrations in fruits (edible part of tomato crops).	Measuring different physical and chemical properties is very difficult.	[29]
	Modeling the behavior of pesticide residues in tomatoes and their associated long-term exposure risks	Tomato	Chlorothalonil, deltamethrin, deltamethrin, alpha-cypermethrin, bifenthrin, Captan, folpet, tebuconazole, triadimenol, triadimenol, metalaxyl-m, chlorpyrifos-methyl, lambda-cyhalothrin	Determining the health effect of pesticides.	An adequate description of pesticides’ degradation and their behavior after application is important in providing inputs for these models.	[30]
	Modeling pesticide residue uptake by leguminous plants: a geocarpic fruit model for peanuts	Peanut plant	Lipophilic and hydrophilic pesticides	This can be used to predict residue concentrations in edible legume seeds.	The pH-dependent physicochemical properties (e.g., soil–water partition coefficient and TSCF) and degradation rate constants of the chemicals need to be refined to improve the simulation analysis.	[31]
	Dissipation kinetics, residue modeling and human intake of endosulfan applied to okra. (Abelmoschus esculentus)	Okra	Endosulfan	To evaluate pesticide residues in crops grown for human consumption, including their isomers and metabolites.	Only pesticide kinetics is considered.	[32]
Aerial parts Analyzer	Dynamic multi-crop model to characterize Impacts of Pesticides in food	Wheat, rice, tomato, apple lettuce, potato.	Tebuconazole, chlorothalonil, carbaryl, chlorpyrifos, azoxystrobin	Determining the presence of pesticides in six types of food crops and characterizing the health impacts of pesticides applied to food crops.	The present model is limited to neutral organic substances, since inorganics require a different consideration of their partitioning behavior.	[10]
	Plant uptake of pesticides and human health: Dynamic modeling of residues in wheat and ingestion intake	Wheat	Tebuconazole	Analyzing the uptake and translocation of pesticides in wheat after foliar spray application and the subsequent intake fractions by humans.	Neutral organic pesticides in the wheat-environment system.	[33]
	New concepts for dynamic plant uptake models	Spring wheat, carrot	Trichloroethene	Predicting chemical fate in the soil–plant–air system when the input pattern is dynamic.	There is inhomogeneity of units in four simultaneous equations.	[12]
	Fruit Tree Model for uptake of organic compounds from soil and air	Apple, peach	Trichloroethene	Predicting transportation of the polar, non-volatile compounds from soil to fruits, while lipophilic, non-volatile compounds accumulate from the air into fruits.	The model is only valid for polar, non-volatile compounds.	[34]
	Fruit Tree Model for uptake of Organic Compounds from soil	Apple	MTBE, benzene, toluene, trichloroethene, and naphthalene	Predicting transportation of the polar, non-volatile compounds from soil to fruits, while lipophilic, non-volatile compounds accumulate from the air into fruits.	The model is strictly limited to neutral compounds; it is not applicable to ions or dissociating compounds.	[35]
	Advances in life cycle Impact assessment of pesticides: Methodological improvements and experimental studies	Greenhouse tomato	Captan	Determining the relative risk level of pesticides even prior to application.	Two pesticides are analyzed only regarding half-life improvement relation.	[13]
	Generic One-Compartment Model for Uptake of Organic Chemicals by Foliar Vegetation	Grass, corn and all vegetables	2, 3, 7, 8 TCDD	One equation is required for calculation of pesticide uptake from soil.	The proposed model is only valid for specific situations and are of limited use.	[26]
	Modeling the exposure of children and adults via diet to chemicals in the environment with crop-specific models	All leafy vegetables, potato, fruit	Benzopyrene. TCDD	Pesticide in food-stuff contributing to daily exposures (excluding fish) can be identified using this.	It overestimates the exposure to the lipophilic compounds.	[36]
	ZHPO-LightXBoost an integrated prediction model based on small samples for pesticide residues in crops	Rice, romaine, lettuce, and cabbage	Any pesticide	Abamectin, Pymetrozine, Trichlorfon, and Tebuconazole.	This study provides specific practical guidance for farmers’ pesticide use and formulation of relevant regulations on pesticide residues.	[37]
	Generalizing routes of plant exposure to pesticides by plant uptake models to assess pesticide application efficiency.	Any plant	Halofenozide, and paraquat	This model is utilized to determine the efficiency of different pesticides and their process of application on plants.	This study does not consider plant growth stages, pesticide application events and timings, and pesticide formulations to optimize pesticide application.	[38]
	Estimating Half-Lives for Pesticide Dissipation from Plants.	Any plant	Any pesticide	Different properties of the plant, pesticide, soil properties, and environmental temperature were used for finding out the half-lives of pesticides in plant.	Pesticide half-lives in leaves and stems are not measured separately.	[39]
**Indirect Analytical Models**						
Water analyzer	SWASH (Surface water scenarios help) Manual 5.3	Cereal, soybean, sunflower, etc.	Any of the pesticides	It includes the Focus Drift Calculator, MACRO, PRZM-3, and TOXWA models. Commonly, it is used to calculate the concentration of pesticides in surface water.	It does not provide model runs; it provides guidance only.	[40]
	Testing the Greenhouse Emission Model (GEM) for Pesticides Applied via Drip Irrigation to Stone Wool Mats Growing Sweet Pepper in a Recirculation System	Sweet pepper	Imidacloprid, pymetrozine	Concentrations of pesticides in surface water and groundwater can be determined by this model.	Incomplete mixing of pesticide gas with water makes a difference to the simulated and measured values.	[43]
	Volatilization of Parathion and Chlorothalonil from a Potato Crop Simulated by the PEARL Model	Potato	Parathion, chlorothalonil	It is used to evaluate the leaching of pesticides to groundwater and drainage.	This model does not consider the part of the dosage that is intercepted by the plants, washed off by rainfall, or the volatilization of pesticides from a surface film and also it is more difficult to obtain rate coefficients for the processes competing with volatilization.	[41]
	Regression Modeling for Monitoring Organochlorine Pesticide Residues	Silver carp fish	Chlorpyrifos, folpet, lindane, pbo, pendimethalin, and tebuconazole	To provide awareness of pesticide distribution in the environment.	The dimensionality of the surface water sample array is insufficient to build adequate mathematical models.	[44]
Soil Analyzer	Numerical and analytical model of pesticide root uptake model comparison and sensitivities	Cereal	Any pesticide	Determination of chemical sorption and degradation input parameters is possible.	Linear sorption models, which are often used in pesticide leaching studies, may yield incorrect results.	[42]
	Development and application of an advanced algorithm for safety management of pesticide residues in agricultural soils: Monitoring of currently used pesticide in upland soils	Citrus, pear, apple, peach, persimmon, grape	116 pesticides	This research established a comprehensive system capable of monitoring, risk assessment, and safety management for pesticides in soil.	Continuous monitoring and evaluation are required.	[45]

**Table 3:** Existing semi-empirical models that estimate residues of pesticides in different parts of plants are shown below.

	Pesticide Model Name/Journal Paper Name	Plant for Experiment	Pesticides Used	Application of the Models	Limitations	Reference
**Direct Effect Models**						
Aerial Parts Analyzer	Considering kinetics of pesticides in plant uptake models: proof of concept for potato	Potato	Thiamethoxam, mepiquat, chlorpyrifos	It evaluates the degradation kinetics of pesticides in plant tissues.	This simplified method may overestimate pesticide residue levels in harvested plants.	[18]
**Indirect Effect Models**						
Water Organism Analyzer	MASTEP (Metapopulation model for Assessing Spatial and Temporal Effects of Pesticides model)	Asellus aquaticus	Any pesticide	The motto of the model is to predict recovery of aquatic invertebrates following pesticide stress.	Some of the parameters of the model that depend on movement and density are dependent on processes that possess insensitivity and sensitivity.	[46]
Water Analyzer	CASCADE	Potato, sugar beet	Any pesticide	It predicts exposure at different locations with catchments and simulates pesticide concentrations in systems of ditches at a scale of the order of 10 km^2^.	The convective transport of solutes in soil by infiltrating water was ignored.	[47]
	GLEAMS (Groundwater Loading Effects of Agricultural Management System)	Corn	Atrazine, cyanazine alachlor, bromide	It simulates pesticide leaching to groundwater.	GLEAMS was not developed as an absolute predictor of pollutant loading.	[48]

**Table 4:** Existing empirical models that estimate residues of pesticides in different parts of plants are shown below.

	Pesticide Model Name/Journal Paper Name	Plant for Experiment	Pesticides Used	Application of the Models	Limitations	Reference
**Direct Effect Models**						
Fruit/Grain analyzer	Dynamics of pesticide uptake into plants: from system functioning to parsimonious modeling	Wheat	Carbaryl, cyromazine	Determination of pesticides in a multi-compartment plant–environment system by mathematical decomposition techniques.	These models are usually restricted to assess impacts from pesticide mass fractions lost from the model’s scope during and after application, thereby ignoring intake of pesticides from the mass directly reaching the target crops.	[49]
	Development and application of a numerical dynamic model for pesticide residues in apple orchards	Apple orchards	Four suitable pesticides for apple	This simulation measures pesticide concentrations in soil and different plant compartments.	Distribution of pesticide in air and its effect on plant is not considered.	[50]
Leaf analyzer	Fate of the organophosphate insecticide, chlorpyrifos, in leaves, soil, and air following application	Purple tansy	Chlorpyrifos	It advances understanding about chlorpyrifos behavior in 79 agricultural environments by conducting a comprehensive investigation into its fate and loss rates post-application.	Predict and understand the emission rates of semi-volatile pesticides from agricultural fields since reliable values are needed.	[51]
Stem analyzer	Lignin and lipid impact on sorption and diffusion of trichloroethylene in tree branches for determining contaminant Fate during plant sampling and phytoremediation	Red-maple tree, silver-maple tree, white pine, tulip, linden	Trichloroethylene (TCE)	Quantifying the roles of lipid and lignin on equilibrium sorption and diffusion in tree branches and bark.	The assumption has not been verified in the field.	[52]
Aerial parts analyzer	Uptake of organic contaminants from soil into vegetables and fruits	Cereal, carrot, lettuce, potato and apple tree	Perchloromethane, trichloroethene	Predicting the uptake of organic contaminants from soil in to vegetables and fruits.	Uncertainty in the predictions of plant uptake due to immense variation in environmental and plant physiological conditions.	[53]
	Modeling pesticides residues	Wheat	Bromo xylene, tebuconazole	Determining the presence of residues in agricultural commodities.	Some factors are not considered due to the lack of descriptive and quantitative methodology.	[4]
**Indirect Effect Models**						
Water analyzer	Dry Deposition and Spray Drift of Pesticides to Nearby Water Bodies	Any plant	Acephate, alachlor, aldicarb, amitrole, etc.	To estimate spray drift of pesticides nearby water bodies.	Dry deposition is limited to the laminar boundary. So, it is difficult to show dry deposition flux for all compounds.	[54]
	Freshwater ecotoxicity assessment of pesticide use in crop production: Testing the influence of modeling choices	Maize, winter wheat, grass, spring barley, rapeseed, and pea	Glyphosate	This model assesses the freshwater ecotoxicity of the pesticide.	If dynamics are to be considered, the relevant data have to be consistently reported.	[17]
Soil analyzer	Effect of sprayer parameters and wind speed on spray retention and soil deposits of pesticides: Case of artichoke cultivar	Artichoke plants	Brilliant sulfoflavine.	It is used to predict pesticide deposition on the foliage and those lost on the soil.	The models tended to reflect the measured data, but with a slight over-prediction, especially for the field measurements due to the limited number of the studied combinations.	[55]
	Crop-specific human exposure assessment for polycyclic aromatic hydrocarbons in Czech soils	Carrot, lettuce, potato, apple tree	Naphthalene, acenaphthylene, acenaphthene, fluorene, phenanthrene, anthracene, fluoranthene, pyrene, chrysene, benz(a)anthracene, benzo(a)pyrene, benzo(e)pyrene, benzo(b)fluoranthene, benzo(j)fluoranthene, benzo(k)fluoranthene, benzo(g,h,I) perylene, indeno(1,2,3-cd)pyrene, dibenz(a,h)anthracene	This model is used to derive rational soil quality standards for a large variety of chemicals with reasonable effort.	Soil attached to root vegetables and potatoes was not considered in this study (careful peeling was assumed).	[56]

**Table 5:** Existing discussion articles that estimate residues of pesticides in plants are shown below.

Pesticide Model Name/Journal Paper Name	Plant for Experiment	Pesticides Used	Application of the Models	Limitations	Reference
**Direct Effect Articles**					
Plant Uptake and Transport Models for Neutral and Ionic Chemicals	Apple, potato	MTBE, benzene, toluene, trichloroethene, and naphthalene	To differentiate between models for neutral compounds, and models for weak and strong electrolytes.	The accuracy of the model for neutral compounds is satisfactory; the prediction of electrolyte behavior inside plants is still quite difficult.	[58]
Organochlorine pesticide residues in plants and their possible ecotoxicological and agri food impacts.	Banana, brinjal, lotus, tomato	γ-HCH (lindane), heptachlor epoxide isomer, dieldrin, endrin, endrin aldehyde and endrin ketone	Determination of pesticides in the non-edible parts of the plant to check the ecotoxicological and agri-food impact.	Evaluation was done only in the non-edible parts of the plants.	[59]
Effects of food processing on pesticide residues in fruits and vegetables: A meta-analysis approach.	Tomato, carrot, potato, etc.	Dimethoate, DDT, endrin, etc.	Determining the effects of various food processing technique on pesticide residues.	Some calculations were performed using a limited number of experiments.	[60]
Life cycle human toxicity assessment of pesticides: Comparing fruit and vegetable diets in Switzerland and the United States.	Lettuce, melon	Captan, mancozeb	It makes the calculation of human health impacts possible.	The uptake from air into specific plant tissues after deposition on leaves or fruits was neglected, although it is known to be an important uptake pathway as most pesticides are directly sprayed on plant surfaces.	[57]
Photodegradation of pesticides on plant and soil surfaces	Apple trees, garden beans, kidney beans rice plants, marrow plants, etc.	Organochlorines (DDT, aldrin, dieldrin and endrin), organophosphorus esters, pyrethroids (the trans- and cis-isomers of 14C-phenothrin), carbamates metolcarb, xylylcarb, and trimethacarb, azoles, etc.	This model can quantify factors controlling photodegradation, together with meteorological factors.	A change in molecular excitation, deactivation, and photodegradation mechanisms are the major drawbacks.	[61]
**Indirect Effect Articles**					
PRIMET (Pesticide Risks in the Tropics to Man, Environment and Trade model)	Non-target arthropod	Imadochlorpid, cypermethrin, epoxiconazole	It is used for calculating the risk of pesticide application to aquatic and terrestrial life. It needs limited parameters, is cost-effective and can be used without specialized training.	The need for specific local data which may be scarce in some areas, and a possible limited ability to capture complex interactions within ecosystems.	[62]
Life cycle impact assessment of pesticides on human health and ecosystems.	Wheat	Chlorothalonil, cyproconazole, hexaconazole, tebuconazole, flusilazole	Determining the impact of pesticides on human health and ecosystems.	The analysis is not based on a real environment, but on a simplified hypothesis.	[9]
Foliar Photodegradation in Pesticide Fate Modeling: Development and Evaluation of the Pesticide Dissipation from Agricultural Land (PeDAL) Model	Chinese cabbage, collard, cotton, kale, orange, potato, purple tansy, and rose	2,4-dichlorophenoxyacetic acid (2,4-D), azadirachtin, chlorothalonil, chlorpyrifos, fenitrothion, and parathion	It is significantly faster, easier, and cheaper than other methods typically used to estimate pesticide fluxes from agricultural fields.	It is currently designed to estimate pesticide dissipation for pesticide that lands on the outer canopy of plants.	[63]

## Data Availability

No new data were created or analyzed in this study.
